# Editorial: Predicting High-Risk Individuals for Common Diseases Using Multi-Omics and Epidemiological Data

**DOI:** 10.3389/fgene.2021.737598

**Published:** 2021-08-18

**Authors:** Debajyoti Chowdhury, Xin Zhou, Bailiang Li, Yuanwei Zhang, William K. Cheung, Aiping Lu, Lu Zhang

**Affiliations:** ^1^Computational Medicine Lab, Hong Kong Baptist University, Kowloon Tong, Hong Kong; ^2^School of Chinese Medicine, Institute of Integrated Bioinformedicine and Translational Sciences, Hong Kong Baptist University, Kowloon Tong, Hong Kong; ^3^Department of Biomedical Engineering, Vanderbilt University, Nashville, TN, United States; ^4^Department of Radiation Oncology, Stanford University School of Medicine, Stanford, CA, United States; ^5^The Chinese Academy of Sciences Key Laboratory of Innate Immunity and Chronic Diseases, Hefei National Laboratory for Physical Sciences at the Microscale, Chinese Academy of Sciences Center for Excellence in Molecular Cell Science, Collaborative Innovation Center of Genetics and Development, School of Life Sciences, The First Affiliated Hospital of University of Science and Technology of China, Hefei, China; ^6^Department of Computer Science, Faculty of Science, Hong Kong Baptist University, Kowloon Tong, Hong Kong

**Keywords:** multi-omics analyses, disease prediction, machine learning, complex diseases, data integration analysis

Physiological data are the reflections of the physiological status of living systems (Terranova et al., 2021). It is precious and preserves meticulous information. Capturing, interpreting, and rationalizing them is imperative for next-generation medicine. Obtaining real-time, patient-centric data have been progressively positioned at the core of digital disruption in healthcare. It promises to deliver an accurate yet early diagnosis, and personalized precision therapy (Esteva et al., 2019). The advent of multi-omics technologies and proficiency in utilizing complex, multi-dimensional biological, epidemiological, and clinical data from bench-side to real-world have significantly steered biomedical research and healthcare practices. With the mounting resources of multi-omics data including transcriptomics, genomics, proteomics, metabolomics, and epigenomics, it becomes challenging to integrate and infer them to insights. However, it is essential in reimagining the scopes of discoveries in predictive healthcare (Boniolo et al., 2021; Ding et al., 2021).

This special issue congregated 15 different studies demonstrating different computational frameworks, algorithms, and methods for inferring multi-omics, high-throughput data for predictive health and early diagnosis of many common diseases. This issue covered different conditions including sleep, gynecological, and oral health, common viral infections, and different cancers including breast cancers (BC), multiple myeloma (MM), stomach adenocarcinoma (SA), esophageal cancer (OC), gastric cancer (GC), and hepatocellular carcinoma (HC).

The majority of the studies published in this topic have introduced diverse methods to predict risks for different cancers (Guo et al.; He et al.; Liu et al.; Pang et al.; Song et al.; Sun J. R. et al.; Sun Z. et al.; Zhao et al.; Zhang et al.; Zhou et al.). Zhou et al. introduced a novel long non-coding RNAs (lncRNAs) based screening method that can indicate risk score for MM. They obtained the raw transcriptome data from Gene Expression Omnibus by performing weighted gene co-expression network analysis (WGCNA) and principal component analysis to identify several risk lncRNAs. Successively, they employed univariate, least absolute shrinkage, and selection operator (LASSO) Cox regression and multivariate Cox hazard regression analysis to identify the reliable targets of the lncRNAs, LINC00996 and LINC00525 to devise a predictive risk score system. These lncRNAs were associated with survival and involved in the occurrence and progression of MM. Similarly, Zhao et al. identified the six-lncRNA signature as a potential prognostic marker to predict disease-free survival of BC patients. Liu et al. introduced an effective multi-gene modeling framework to predict the overall prognosis of heterogenous SA including their signature mutations. They collected two independent SA cohorts with both genetic profiling and clinical follow-up data to investigate the association between the somatic mutations and prognosis. Guo et al. identified a practical and robust nine-gene prognostic model based on an immune gene dataset. Immune-related genes (IRGs) are crucial contributors to the development of EC. The authors studied the transcriptome data and matched it with the clinical data of OC patients from The Cancer Genome Atlas (TCGA) database. GEPIA2.0 was employed to analyze 4,094 differentially expressed prognostic genes among the 286 normal from Genotype-Tissue Expressions (GTEx) and 182 TCGA samples. Then, they used Clusterprofiler for Gene Ontology annotations and Kyoto Encyclopedia of Genes and Genomes enrichment analysis and performed joint Cox regression analysis to study candidate prognostic biomarkers for OC. Relying on this, they estimated the risk scores of each patient from the expressions of differentially expressed IRGs and the regression coefficient from the regression model.

Sun J. R. et al. focused on alternative splicing (AS) and flagged the AS events as a reliable biomarker for the prognosis of OC. They constructed the splicing factors-AS correlation networks to offer new insights in identifying the potential regulatory mechanisms associated with OC development. In the second study by this team, genomic scores (GS) were calculated based on Genome-Wide Network Analysis to predict the survival in GC (Sun Z. et al.). Their multivariate analysis revealed a GS strategy as a novel prognostic factor that comprises 7 miRNAs, 8 mRNA, and 19 DNA methylation sites.

The power of machine learning models have emerged in the study by He et al. Sequencing-based identification of tumor tissue-of-origin (TOO) is critical for patients with cancers of unknown primary lesions. There has always been a probability of misdiagnosis. To avoid those issues, He et al., developed a machine learning model using the expression of a 150-gene panel to infer the tumor TOO for 15 common solid tumor cancer types, including lung, breast, liver, colorectal, gastroesophageal, ovarian, cervical, endometrial, pancreatic, bladder, head and neck, thyroid, prostate, kidney, and brain cancers. They studied 7,460 primary tumor samples across those 15 cancer types and employed the Support vector machines based recursive feature elimination algorithm to perform the feature selection and classification modeling on gene expression data. It designated 154 out of the 11,925 genes with distinct biological significance. Thus, they elucidated a robust classifier on gene expression data to predict TOO-based accurate reclassifications of cancer types which were supplemented with clinical examination.

Zhang et al. introduced an interesting method relying on miRNA-based nomogram to predict distal lung metastasis of BC. They acquired miRNA and clinicopathological data from the Molecular Taxonomy of Breast Cancer International Consortium (METABRIC) and screened out 8 miRNAs as highly relevant to lung metastasis of BC patients. They used the limma package to distinguish miRNAs annotated within the METABRIC dataset and differentially expressed miRNAs (DEMs). They employed LASSO regression to select the most suitable predictive miRNAs from the 16-lung metastasis-related DEMs and formulated a risk-score prediction tool relying on 8-miRNAs for predicting lung metastasis status of BC patients in the training set. Then, they used univariate and multivariate logistic regression analysis to determine the proficiency of those 8 miRNAs as predictors and employed decision-curve analysis to test its clinical applicability. Song et al. investigated a vital direction to identify the hub genes associated with HC. Using a Robust Rank Aggregation method combined with WGCNA, they constructed a clinically relevant prediction model to uncover the complex biological mechanisms of HC.

Sleep is one of the most neglected public health concerns. Sambou et al. instituted a large study comprising the big data obtained from 328,850 participants to endorse a data-driven decision on the associations of the quality of sleep and the healthier life span.

Implantation failure (IF) is one of the recurring issues in assisted pregnancy (Busnelli et al., 2021). Thin endometrium (TE) is a critical factor in IF. mRNA-miRNA cross-talks have been repeatedly flagged as one of the essential etiologies for IF. Xu, B et al., reconstructed integrative transcriptional regulatory networks based on the miRNA-mRNA expression profiles in the TE and normal endometrium tissue obtained from 8 patients (Zong et al.). It involved the miRNA sequence analysis using the DeAnnIso tool (Zhang et al., 2016). They employed Solexa CHASTITY and Cutadapt pipeline to process mRNA sequence data and identified multiple hub genes by constructing the miRNA–mRNA regulatory networks that illuminate new insights underpinning the TE formation (Zong et al.). Huang et al. studied single-cell transcriptional profiles to identify the impact of sex and age on the gene expression of endothelial cells. The transcriptomes of endothelial cells from 5 organs, heart-aorta, fat, lungs, limb, muscle, kidney of the mouse were analyzed. It discovered that older mice had increased expressions of genes involved in inflammation in endothelial cells, which may contribute to the development of chronic, non-communicable diseases like atherosclerosis, hypertension, and Alzheimer's disease with age.

Another study focused on host-pathogen interactions and devised oligoadenylate synthetases-like (OASL) as a potential biomarker for early detection of flu-mediated acute respiratory infection (ARI) cases (Li et al.). This study was aimed to distinguish a strong single-gene biomarker with a superior diagnostic accuracy by using integrated bioinformatics analysis with XGBoost, a feature selection method relying on recursive feature elimination with cross-validation (Li et al.). They analyzed transcriptome profiles to reconstruct a co-expression network by employing WGCNA to identify the OASL as a hub gene for ARI. Pang et al. applied random forest to predict dental caries risks among teenagers. They constructed the caries risk prediction model that serves as an easy, accessible community-level tool to identify individuals with high caries risk.

All of the research articles published under this topic introduced the state-of-the-art technologies employed on multiplexed physiological data. It offers a newer perspective on the early diagnosis of different diseases using data-driven approaches. We anticipate it will be impactful in accelerating the scopes in predictive healthcare research and applications.

## Author Contributions

This editorial was designed by DC and LZ, written by DC, edited and revised by LZ, XZ, BL, and YZ, and supported by WC and AL. All authors made a direct and intellectual contribution to this topic and approved the article for publication.

## Conflict of Interest

The authors declare that the research was conducted in the absence of any commercial or financial relationships that could be construed as a potential conflict of interest.

## Publisher's Note

All claims expressed in this article are solely those of the authors and do not necessarily represent those of their affiliated organizations, or those of the publisher, the editors and the reviewers. Any product that may be evaluated in this article, or claim that may be made by its manufacturer, is not guaranteed or endorsed by the publisher.
